# On the Application of Concentrated Nitric Acid to Irritable Ulcers of the Face, Nose, &c.

**Published:** 1824-02

**Authors:** Charles Averill

**Affiliations:** Surgeon, Cheltenham; Member of the Royal College of Surgeons.


### Art. II.—*On the Application of concentrated Nitric Acid to irritable Ulcers of the Face, Nose, &c.* By Charles Averill, Surgeon, Cheltenham; Member of the Royal College of Surgeons.

Having seen, in the second part of the last volume of the Medico-Chirurgical Transactions, a paper of Mr. Earle's, on Local Irritation and on Diseases of the Integuments of the Nose and Face, I am led to offer the following observations and cases to your notice.

That those indolent ulcers, which from their irritable nature are called *Noli me tangere,* are, in the first instance, dependent on some constitutional derangement, seems most probable; but numerous cases demonstrate that constitutional treatment alone is not sufficient for their cure, and that local remedies, of the most active kind, are absolutely requisite.

As far as I have observed, these irritable sores are most commonly found among the lower ranks of society, in young persons, whose scrofulous constitution, and white and delicate skin, is peculiarly liable to suffer from exposure to the vicissitudes of climate; under those circumstances of uncleanliness and moisture, which, from their disposition to catarrhal affections, can scarcely be prevented in persons of their habits. The principal of the local treatment of these cases, as justly stated by Mr. Earle, is, that the parts involved in the diseased action should be artificially removed. He proposes that this should be done by the scalpel, the potassa fusa, or the arsenical paste. In several cases, it has appeared to me that the application of concentrated nitric acid to the ulcerated surface is better adapted for its removal than either of those three methods. The sores are often so numerous or so extensive, or they implicate parts of such a nature, as to render their excision uncertain, or even unjustifiable. The depth to which sloughing is produced by any application of the potassa fusa is not to be known, from the discoloration it immediately causes; its action is therefore uncertain,—perhaps in part owing to the different degrees of purity of the preparation: this is also a much more painful caustic than the nitric acid, even when both are pure.

The employment of arsenic has been known to produce fatal effects by its absorption, when applied to a considerable surface: whereas, any escharotic, applied to a part only of a large ulcer, has been found to render it eventually worse; the newly deepened surface being always exposed to the irritating discharge from the diseased, and likely to resume its morbid character. The arsenic applied to a number of small ulcers successively, is a tedious remedy: to one, however, of a moderate size it may be used with success.

The two following cases exemplify the good effect of the nitric acid.

Elizabeth Truby, of Charlton, Worcestershire, aged seventeen years, of a fair and delicate complexion, applied to me, December the 14th, 1820, her face having the appearance here described:—the tip and alæ of the nose were much swollen, ulcerated, and partly incrusted by scabs of a dirty yellow colour; the surrounding integument was of a purple hue, which extended up the nose, partly over the left cheek, and to the upper lip; in the substance of which were two irregular scabby ulcers, giving it the appearance of a double hair-lip. From the whole ulcerated surface, as well as from the interior of the nose, there was a copious discharge of thick white matter, which smelt offensive. The disease had existed four years, first making its appearance in the form of a small pimple, which ulcerated and gradually extended, till it bore the appearance above described. She had been one year and a half a patient in an infirmary, where she had taken a considerable quantity of medicine, and used various applications to the parts. It was three months since she had any menstrual discharge, and that to a very trifling extent, and for the first time. In other respects, her general health appeared tolerably good. I ordered her, as an alterative medicine, to take a tea spoonful of the following drops three times a day, in a little water:—℞. Hydr. Oxymuriatis, gr. ij.; Spir. Ætheris Nit. ʒij. M.; and to keep the sores constantly wet with lint dipped in the following lotion:—℞. Liq. Arsenicalis, ʒiij.; Aquæ pur. ℔j. M.

December 18th.—The ulcers were rather cleaner, and of a less irritable appearance. The medicine at first made her sick, and occasionally purged her; or, to use her own expression, "searched her a good deal."

Dec. 21st.—The menstrual discharge had returned, and the discharge from the ulcers diminished, and become of a less offensive nature. She had two or three stools daily.

Dec. 28th.—No improvement, but the contrary. The discharge had increased, and the ulcers resumed their irritable and foul appearance. She was ordered to continue the drops; and, in order to destroy the surfaces of the ulcers, to dress them twice a-day with the following ointment:—℞. Oxyd. Arsen. ʒss; Cerat. Cetacei, ʒvj. M. This application did not produce a slough, but excited a little inflammation around the ulcers, which became rather larger, as well as a serous effusion in the cellular membrane under the eyes.

January 4th, 1821.—Being fearful of increasing the quantity of arsenic, I painted the ulcerated surfaces over with strong nitric acid, and directed them to be covered with lint wet in the following—℞. Acid. Nitrici, gr. l.; Aquæ puræ, ℔ij. M.

Jan. 8th.—The application of the strong acid had caused sloughs over every ulcer, and very considerable inflammation in the surrounding parts, which were much swollen, and so irritable, that the application of the diluted acid could no longer be borne; a warm poultice of oatmeal and beer was, therefore, ordered to be applied over the greater part of the face.

Jan. 13th.—The inflammation and swelling had diminished, and a small slough had separated from the lip, leaving a healthy granulating surface. She was ordered to continue the poultice; took no medicine.

Jan. 15th.—Another small slough had separated from the lip, which was now quite clean, and granulating healthily. Ordered to continue the poultice, and take one of the following powders twice a-day:—℞. Hydr. c. Creta, ʒj.; Sodæ Subcarbon. ℥ss. M. et div. in ch. xij.

Jan. 17th.—The sloughs had separated from the nose, leaving healthy granulations, and the ulcers of the lip had nearly healed. She was ordered to leave off the poultice, and again apply the nitric-acid wash, with a view of forwarding the process of cicatrization.

Jan. 22d.—Scabs had formed over the sores on the nose; the poultice was therefore ordered to be applied, instead of the wash. The lip was quite healed, and she was desired to take one of her powders every night.

February 5th.—The outer surface of the nose was quite healed, but there was still a very considerable discharge from its interior; to diminish which, she was directed to apply the following astringent lotion, both externally and internally, by means of plugs of lint:—℞. Zinci Sulphat. ʒij.; Plumbi Superacet. ʒj.; Aquæ puræ, ℔j. M.

Feb. 19th.—The discharge was much diminished in quantity, and the size of the nose considerably reduced. She was ordered to continue the powders and the lotion, but to weaken the strength of the latter by adding to it another pint of water. She persisted in the use of both for a month, when, the discharge having ceased, she left them off.

On the 1st of May following I saw her, when she had no other appearance of disease than that of the skin covering the lower part of the nose, and the upper lip, being of a deeper hue than the surrounding integument.

The following case, of which I made notes at the time, was a patient in Guy's Hospital, under the care of Sir Astley Cooper. It shows, in the most satisfactory manner, the excellent effects of nitric acid as a caustic.

Joseph Hester, aged eighteen years, was admitted into Luke's ward, Guy's Hospital, under the care of Sir Astley Cooper, on the 11th of July, 1821, with several superficial scabby ulcers situated on the sides of the face and nose, the largest being rather bigger than a shilling. They had existed for nine years, during which time he had almost continually been making use of some application or other, and had tried a great variety of these, as well as the exhibition of medicine, without any apparent benefit. He had been recommended to try the effect of sea-air, and for this purpose had resided fifteen months by the sea-side; at which time he was ordered the occasional—indeed, rather frequent,—use of the bath, together with those constitutional remedies which the nature of the case might seem to require. This plan of treatment was, however, unavailing, and the complaint consequently remained undiminished. At the time of his admission into Guy's, he was directed to take the following draught three times a-day:—℞. Sol. Arsenicalis, gtt. v.; Decoct. Cincho, ℥j. M.; and a poultice was ordered to be applied over the incrustations covering the ulcers.

On the 17th of July, the scabs being removed, the parts were painted over with undiluted nitric acid, and again poulticed. The application of the acid produced severe inflammation, attended with very considerable swelling of the face, pain in the head, and fever; for which he was cupped on the back of the neck, and ordered an opening medicine, consisting of Epsom salts and mint julep, the effect of which was a very speedy removal of the pain; the inflammation quickly subsided, and the swelling gradually diminished. On the 31st, the sloughs formed by the acid having separated, the zinc lotion was applied, the parts looking extremely healthy and disposed to cicatrize. On the 7th of August, the lotion was repeated, the sores looking very healthy, and having every prospect of being speedily healed.

August 10th.—Scarcely any appearance of disease remained. Sir Astley, however, deemed another application of the strong acid requisite, in order more effectually to eradicate this long-standing evil. It was applied, on the 11th, to the several parts where the ulcers had been situated, and afterwards a poultice. On the 13th, the face was much swollen, but there was no fever present to render medicine requisite: the common aperient mixture of the house, however, was occasionally taken. The poultice was continued till the 28th, when the sloughs had all separated; the edges of the sores appeared tumidly thick; the zinc lotion was ordered to be applied as before. From the time the lotion was applied the sores healed astonishingly fast. By the 3d of September they were all cicatrized, and there only remained a little swelling under each eye. The next presenting day he was ordered to be discharged.

